# Urological diagnostics based on kidney stone detection in CT imaging using YOLOv8 deep learning framework

**DOI:** 10.3389/fmed.2026.1702159

**Published:** 2026-03-18

**Authors:** Yuguang Ye, Kavimbi Chipusu, Liuying He, Suo Shen, Jianlong Huang

**Affiliations:** 1 School of Mathematics and Computer Science, Quanzhou Normal University, Quanzhou, China; 2 Fujian Provincial Key Laboratory of Data-Intensive Computing, Quanzhou Normal University, Quanzhou, China; 3 Key Laboratory of Intelligent Computing and Information Processing (Quanzhou Normal University), Fujian Province University, Quanzhou, China; 4 Division of Biomedical Engineering, Department of Mechanical Engineering, University of Saskatchewan, Saskatoon, SK, Canada; 5 Department of Urology, First People's Hospital of Fuyang, Hangzhou, China

**Keywords:** clinical decision support, kidney stones, medical image analysis, object detection, computed tomography, YOLOv8

## Abstract

**Introduction:**

Kidney stone disease is a common urological condition requiring timely detection to prevent complications. Non-contrast computed tomography (CT) is the gold standard for detecting renal calculi, but manual interpretation is time-consuming and subject to variability.

**Methods:**

This study evaluates four deep learning object detection models—YOLOv8, YOLOv5, Faster R-CNN, and RetinaNet—for automated kidney stone detection in CT images. A dataset of 4,000 annotated CT slices from 170 patients was used. Performance was evaluated using mAP@0.5, precision, recall, false positive and false negative rates, and inference speed.

**Results:**

Faster R-CNN achieved the highest localization accuracy (mAP@0.5 = 0.93), while YOLOv8 demonstrated the best balance between accuracy (mAP@0.91) and computational efficiency, achieving real-time inference at 65 FPS.

**Discussion:**

The results highlight the trade-off between detection accuracy and processing speed across architectures. YOLOv8 provides an optimal balance for clinical implementation due to its strong performance and real-time capability.

## 1 Introduction

**Figure 1:**
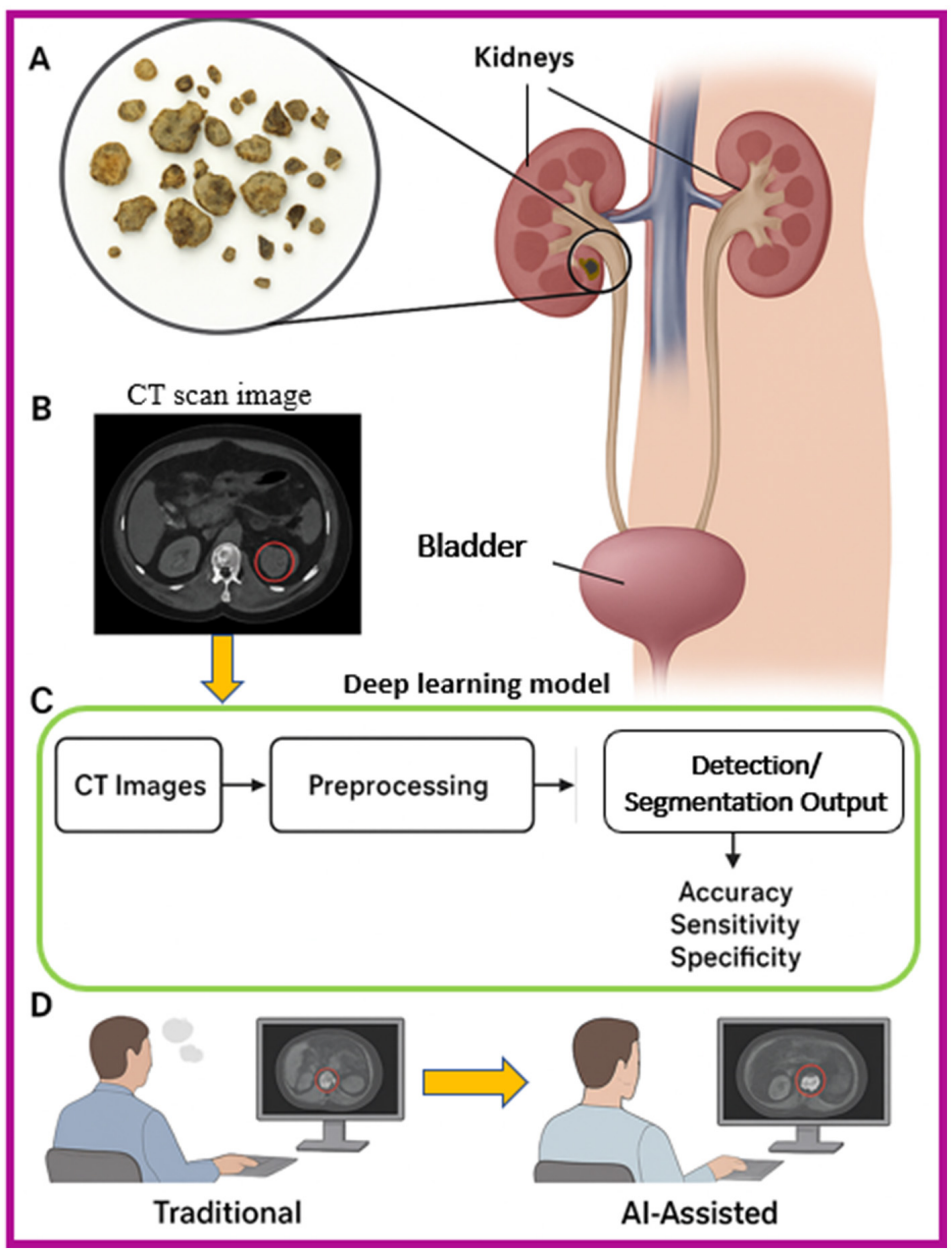
Schematic overview of kidney stone detection and AI optimization, **(A)** Anatomical illustration of the urinary system with kidney stones, **(B)** CT scan image highlighting a kidney stone, **(C)** Deep learning workflow for detection, including preprocessing and segmentation, **(D)** Comparison between traditional radiologist interpretation and AI-assisted detection for improved diagnostic performance.

Kidney stones (1, 2), medically referred to as renal calculi, are crystalline mineral formations that develop within the renal parenchyma or the collecting system (Figure 1). Their incidence has increased globally, affecting approximately 10–15% of the population at some point in their lives. Left untreated or undetected, kidney stones can result in severe pain, obstruction of the urinary tract, hematuria, infections, and, in extreme cases, irreversible renal damage. The burden of urolithiasis has made its early detection and accurate localization a crucial objective in urological diagnostics and treatment planning. The primary imaging modality for kidney stone detection is non-contrast CT, which provides high-resolution cross-sectional images capable of revealing even minute calculi. CT is preferred due to its high sensitivity and specificity for stone detection, outperforming ultrasound and plain abdominal radiography, particularly in obese patients or when the stones are radiolucent. However, despite the strengths of CT imaging, the manual interpretation of thousands of CT slices per patient by radiologists remains a labor-intensive, time-consuming task that is susceptible to inter- and intra-observer variability. Moreover, smaller stones (<3 mm) can be overlooked, especially in high-throughput emergency settings.

In recent years, Artificial Intelligence (3), particularly deep learning (4–6), has shown immense promise in revolutionizing medical image analysis. Deep convolutional neural networks, which can learn hierarchical feature representations from raw pixel data, have demonstrated state-of-the-art performance in tasks such as segmentation, detection, and classification of anatomical structures and pathological entities. In urology (7–9), deep learning applications have emerged for prostate segmentation (10), bladder cancer detection (11, 12), and now, increasingly, for automated detection of renal calculi (13). One of the most practical approaches within DL for object localization is object detection, where the algorithm learns not only to classify an image but to identify and localize specific features or lesions within it using bounding boxes. This is particularly relevant for detecting kidney stones, which may appear as small, dense regions with high Hounsfield Unit (HU) values embedded in a background of varying tissue densities.

This study aims to explore and compare the performance of modern object detection algorithms, YOLOv8 (14, 15), YOLOv5 (16–18), Faster R-CNN (19), and RetinaNet, on the task of kidney stone detection in abdominal CT scans. These models have achieved notable success in general computer vision applications and are increasingly adapted for medical imaging tasks due to their accuracy and speed. YOLOv8, the latest evolution in the YOLO (You Only Look Once) family, offers real-time detection capabilities with improved small object detection performance due to its updated architecture and efficient design. On the other hand, Faster R-CNN, and RetinaNet represent two-stage detection models, which first generate region proposals and then classify them, often resulting in superior accuracy at the cost of slower inference speeds. We seek to evaluate their detection accuracy, precision, recall, and inference speed, while assessing their feasibility for deployment in real-world clinical environments. The goal is not only to determine the most effective model for this specific use case but also to lay the groundwork for integrating AI tools into diagnostic radiology workflows, potentially aiding radiologists in making faster, more accurate decisions.

## 2 Related works

Automated analysis of non-contrast CT for urolithiasis has progressed from slice-level classification to full object detection and volumetry. Early demonstrations used cascaded CNNs to flag urinary stones on unenhanced CT with high discrimination (AUC≈0.95), establishing feasibility for emergency workflows but without precise localization or volumetrics (20). Subsequent studies advanced toward end-to-end detection and measurement. Elton et al. (21) developed a clinically oriented system that segments and quantifies stones on abdominal CT, showing robust detection and automated volume estimation, capabilities critical for treatment planning, and follow-up. Coronal-slice approaches have reported strong performance for automated detection, highlighting the value of leveraging anatomical priors (e.g., kidney-centric views) to reduce false positives from vascular or bowel calcifications (22). Feature-engineering and transfer-learning approaches have explored Darknet-derived and ResNet-based representations for CT stone detection and classification, but these typically operated at image- or slice-level, limiting clinical utility when precise bounding boxes are required (23).

More recently, object-detection frameworks have become prominent. A customized YOLOv5 model with squeeze-and-excitation modules achieved improved mAP and throughput on CT stone detection, underscoring the promise of single-stage detectors for small, high-contrast targets embedded in complex backgrounds (24). Ensemble detection strategies combining multiple CNN backbones have been proposed to improve robustness, though at the expense of computational cost and deployment simplicity. Complementary work demonstrated that YOLO-family detectors can not only identify urolithiasis on CT but also compute stone metrics such as volume and density in real time, outperforming clinicians in speed while maintaining high diagnostic accuracy, evidence that modern single-stage detectors are maturing toward workflow integration (25).

Beyond pure detection, broader reviews have summarized imaging-based deep learning across kidney diseases, positioning stone detection within a wider AI ecosystem in nephrology and urology (26). Related studies extend the scope further: veterinary CT work demonstrates cross-species generalizability of stone detection pipelines, while radiomics–deep learning integration on CT urography has been applied to differentiate medullary sponge kidney–associated stones from other stone phenotypes, both indicative of growing methodological breadth and translational interest around stone characterization on CT (27, 28). Collectively, the literature indicates that (i) CT remains the dominant substrate for AI-assisted stone detection; (ii) single-stage detectors (YOLO variants) increasingly balance accuracy and speed for real-time use; and (iii) comprehensive benchmarking across architectures (YOLOv5/YOLOv8 vs. Faster R-CNN/RetinaNet) on standardized datasets remains limited, motivating the multi-model evaluation presented in this study.

## 3 Methodology

This section outlines the dataset used, preprocessing steps applied to the data, and the object detection models evaluated in this study for kidney stone detection in abdominal CT images, with a specific focus on accommodating pre- and post-surgery patient groups scanned on the same axial plane.

### 3.1 Model architecture

**Figure 2:**
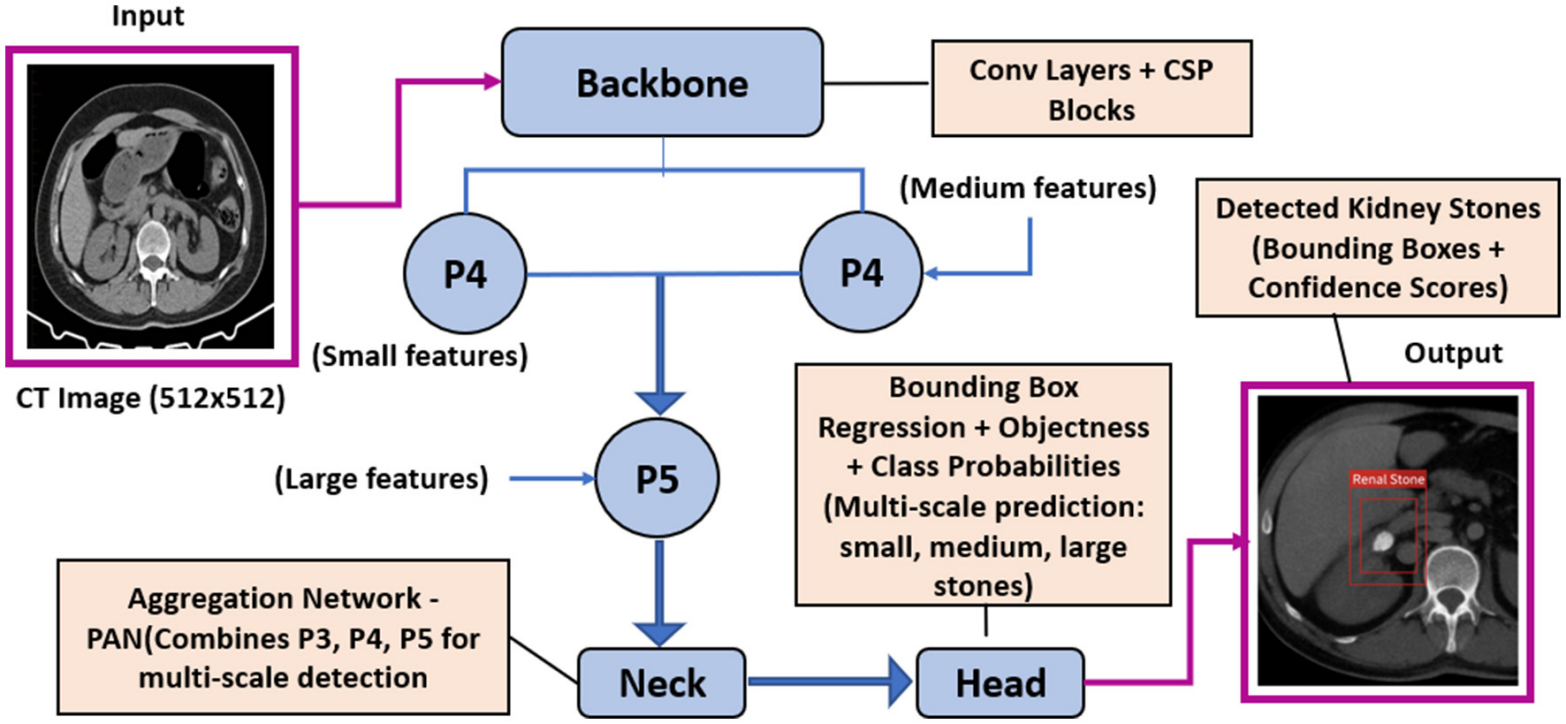
Schematic architecture of the YOLOv8 model for kidney stone detection in abdominal CT images, illustrating the flow from input image through the backbone, multi-scale feature extraction via the neck (PAN), and prediction head generating bounding boxes and confidence scores for stones of varying sizes.

The YOLOv8 model employed in this study is a state-of-the-art single-stage object detection network designed for high accuracy and real-time performance. Its architecture is composed of three main components: the Backbone, Neck, and Head. The Backbone extracts hierarchical feature representations from input CT images using convolutional layers, residual connections, and Cross Stage Partial (CSP) modules, which enhance gradient flow and preserve spatial information essential for detecting small kidney stones. The Neck module aggregates features from multiple scales using a Path Aggregation Network (PAN), enabling the model to combine fine-grained details with high-level contextual information for improved localization across stones of varying sizes. The Head module performs object detection by predicting bounding box coordinates, objectness scores, and class probabilities; YOLOv8 utilizes a decoupled, anchor-free design that directly regresses object locations, simplifying training, and improving generalization. Additionally, the architecture incorporates advanced strategies such as data augmentation, adaptive loss functions, and focus mechanisms that enhance sensitivity to small, high-contrast targets typical of renal calculi. This design allows YOLOv8 to achieve a balance between detection precision and computational efficiency, making it well-suited for deployment in real-time clinical CT workflows. Specifically for the task of detecting small renal calculi, the model's Path Aggregation Network (PAN) facilitates effective multi-scale feature fusion, combining fine-grained details necessary for sub-millimeter targets with high-level contextual information. Furthermore, its anchor-free detection head simplifies the prediction process by directly regressing object centers, which is advantageous for detecting small objects that may not align well with predefined anchor box sizes. The overall architecture is illustrated in Figure 2.

### 3.2 Dataset

The dataset comprised 4,000 non-contrast axial CT slices obtained from 170 patients diagnosed with renal and ureteral calculi. These scans were drawn from the First People's Hospital of Fuyang radiology archive, under approved ethical clearance. To capture different clinical stages, the patients were grouped into: Pre-surgery cases, where CT scans showed calculi of varying size and morphology, often accompanied by hydronephrosis or perinephric exudation. Examples include nodular high-density foci in the left renal calyx (≈6 mm), proximal ureteral stones with upstream dilation, and large distal ureterovesical junction stones up to 14.9 mm in length. Post-surgery cases, including patients who underwent ureteroscopic lithotripsy or stent placement. These scans frequently displayed indwelling double “J” stents, with or without residual high-density foci in the renal or ureteral region, enabling evaluation of residual, recurrent, or fragmented calculi.

All CT images were originally acquired as DICOM sequences and subsequently anonymized to protect patient identity. To ensure compatibility with deep learning frameworks, the DICOM files were converted to PNG format while preserving spatial resolution and contrast characteristics essential for stone visibility. Annotation was performed by three board-certified radiologists. Stones were manually delineated with bounding boxes, with consensus labeling applied to reduce inter-observer variability. Stone identification was based on Hounsfield Unit (HU) thresholds, morphology, and anatomical context within the renal parenchyma, collecting system, or ureter. The dataset was split at the patient level into training, validation, and test subsets, ensuring that images from a single patient were not shared across subsets. This stratification prevented data leakage and improved the generalizability of model performance. Representative cases in the dataset included: Left-sided proximal ureteral stones (≈6–8 mm) with upstream hydronephrosis and perinephric stranding, Bilateral renal calculi with accompanying hydronephrosis, Large ureterovesical junction stones (up to 14.9 mm) with severe upstream obstruction, Post-surgical scans with indwelling double “J” catheters, used to monitor clearance or recurrence of calculi. This diverse clinical spectrum ensured the model was trained and validated on both straightforward and complex cases, including recurrent stones, bilateral involvement, and postoperative follow-up conditions.

This diverse clinical spectrum ensured the model was trained and validated on both straightforward and complex cases, including recurrent stones, bilateral involvement, and postoperative follow-up conditions. To quantitatively summarize the dataset's heterogeneity and justify its use for robust model training, the distribution of key stone characteristics is provided in Table 1. The ground truth annotations were established through a consensus process involving three board-certified radiologists. To ensure annotation reliability, the inter-observer agreement was quantified prior to consensus. The mean Intersection over Union (IoU) between the independent initial bounding boxes from the three radiologists was 0.86 ± 0.07, indicating a high level of initial agreement and supporting the clinical consistency of the final consensus labels used for training.

**Table 1:** Distribution of kidney stone characteristics in the dataset (*n* = 4,000 slices).

**Characteristic**	**Category**	**Approximate percentage/ count**
Stone Size	<3 mm	35%
	3–5 mm	45%
	>5 mm	20%
Stone location	Renal parenchyma/calyces	60%
	Proximal/mid ureter	25%
	Distal ureter/UVJ	15%
Clinical context	Pre-surgery (with obstruction/stranding)	65%
	Post-surgery (with stents/residual fragments)	35%
Laterality	Unilateral	70%
	Bilateral	30%

The dataset was rigorously split at the patient level to prevent data leakage. Patients were randomly assigned to the training (70%, ~2,800 slices from 119 patients), validation (15%, ~600 slices from 26 patients), and test (15%, ~600 slices from 25 patients) sets, ensuring all slices from a single patient were contained within a single split. Stratification was performed to maintain similar distributions of pre- and post-surgery cases across all subsets.

### 3.3 Pre-processing

Pre-processing was a critical step to standardize and enhance the input data for model training and evaluation, applied uniformly across both pre- and post-surgery groups to ensure data consistency. All images underwent windowing normalization, resizing, intensity normalization, and data augmentation as detailed in Table 2. These augmentations were designed to mimic real-world imaging diversity and prevent overfitting, applied equally to pre- and post-surgery images to maintain unbiased training.

**Table 2:** Summary of pre-processing and augmentation parameters.

**Pre-processing step**	**Parameter/ description**	**Value/ range**
Windowing normalization	Window level	350 HU
	Window width	1,000 HU
Image resizing	Target dimensions	512 × 512 pixels
Intensity normalization	Pixel intensity range	[0, 1]
Data augmentation:	Rotation	±15 degrees
	Gaussian noise (standard deviation, σ)	0.01 to 0.05
	Contrast adjustment	±20%
	Horizontal flipping	Applied where anatomically appropriate

### 3.4 Models evaluated

To evaluate the effectiveness of deep learning models in detecting kidney stones in both pre- and post-surgery CT images, four state-of-the-art object detection architectures were selected, each representing different trade-offs between detection accuracy and computational speed. The selected models, their types, and key characteristics are summarized in Table 3. All models were implemented using the PyTorch framework and Ultralytics libraries. Training was conducted on NVIDIA Tesla V100 GPUs, with early stopping based on validation loss to prevent overfitting. Hyperparameters, including learning rate, batch size, number of epochs, and anchor box scales, were optimized via grid search. Model evaluation metrics included mean Average Precision (mAP) at an Intersection over Union (IoU) threshold of 0.5, precision, recall, and inference speed measured in frames per second (FPS). Where clinically relevant, performance was stratified and compared between pre- and post-surgery groups to assess model robustness across differing anatomical conditions.

**Table 3:** Summary of object detection models evaluated in this study.

**Model**	**Type**	**Description**
YOLOv8	One-stage	The latest iteration of the YOLO (You Only Look Once) family, offering improved small object detection, anchor-free design, and real-time performance suitable for clinical use.
YOLOv5	One-stage	A widely adopted version known for stability, speed, and ease of deployment, serving as a baseline for model comparisons.
Faster R-CNN	Two-stage	A high-precision, region proposal-based detector excelling at identifying small and complex objects, albeit with higher computational cost.
RetinaNet	One-stage	Combines one-stage speed with accuracy improvements via focal loss, particularly effective for detecting small lesions and calcifications in medical images.

All models were implemented using the PyTorch framework and Ultralytics libraries. Training was conducted on NVIDIA Tesla V100 GPUs. Early stopping based on validation loss was used to prevent overfitting. Hyperparameters, including learning rate, batch size, number of epochs, and anchor box scales—were optimized via grid search. Model evaluation metrics included mean Average Precision (mAP) at an Intersection over Union (IoU) threshold of 0.5, precision, recall, and inference speed measured in frames per second (FPS). Where clinically relevant, performance was stratified and compared between pre- and post-surgery groups to assess model robustness across differing anatomical conditions.

#### 3.4.1 Hyperparameter tuning and optimization

For a fair and optimized comparison, all models underwent hyperparameter tuning via a grid search on the validation set. For YOLOv8, key parameters included the initial learning rate (tested range: 1e-3 to 1e-5, optimal: 1e-4), batch size (optimized to 16 for our NVIDIA Tesla V100 GPU), and training duration controlled by early stopping (patience = 50 epochs). Compared to training with the model's default parameters, this optimization process yielded an approximate 3–5% relative improvement in mAP@0.5 on our validation set, increasing performance from ~0.87 to our final reported 0.91, while also stabilizing the training convergence. Similar grid search procedures were applied to identify optimal learning rates and batch sizes for YOLOv5, Faster R-CNN, and RetinaNet.

## 4 Results

This section presents the evaluation outcomes of the four object detection models on the test set of the kidney stone dataset.

### 4.1 Quantitative evaluation

To assess the detection performance of each model, we evaluated them on the test set using four standard metrics: mean Average Precision at IoU threshold 0.5 (mAP@0.5), precision, recall, and inference speed measured in frames per second (FPS). Precision and recall were computed using Equations 1 and 2, respectively, where true positives (TP), false positives (FP), and false negatives (FN) were obtained from the model outputs. These metrics reflect each model's ability to correctly identify stones while minimizing incorrect detections. To further interpret detection reliability, the false positive rate (FPR) and false negative rate (FNR), given in Equations 3 and 4, respectively, were also considered in subsequent analysis. Intersection over Union (IoU), a core measure of localization accuracy, was calculated as shown in Equation 5, evaluating how well the predicted bounding boxes overlapped with the ground truth annotations. The overall detection quality was summarized using mAP@0.5 (Equation 6), which averages the precision across recall levels for a fixed IoU threshold of 0.5. Finally, model efficiency was assessed via inference time, which is inversely related to FPS as shown in Equation 7.


$$\begin{array}{l} \text{Precision}=\;\frac{TP}{TP+FP} \end{array}$$ (1)

$$\begin{array}{l} \text{Recall}=\;\frac{TP}{TP+FN} \end{array}$$ (2)

$$\begin{array}{l} \text{FPR}=\;\frac{FP}{FP+TN} \end{array}$$ (3)

$$\begin{array}{l} \text{FNR}=\;\frac{FN}{TP+FN} \end{array}$$ (4)

$$\begin{array}{l} \text{IoU}=\;\frac{Area\;of\;Overlap}{Area\;of\;Union} \end{array}$$ (5)

$$\begin{array}{l} \text{mAP@}0.5=\;\frac{1}{N}\overset{N}{\underset{t1}{\sum }}AP_{i} \end{array}$$ (6)

$$\begin{array}{l} \text{FPS}=\;\frac{1}{Interfernec\;Time\;per\;Image\;\left(in\;seconds\right)} \end{array}$$ (7)

In addition to the standard mAP@0.5, we computed the more comprehensive mean Average Precision over IoU thresholds from 0.5 to 0.95 (mAP@0.5:0.95), which provides a stricter assessment of localization precision across varying degrees of overlap. Table 4 summarizes the results, highlighting the trade-offs between detection accuracy and real-time processing capabilities among the models.

**Table 4:** Performance comparison of detection models on the kidney stone dataset.

**Model**	**mAP@0.5**	**mAP@0.5:0.95**	**Precision**	**Recall**	**FPS**
YOLOv8	0.91	0.68	0.89	0.92	65
YOLOv5	0.88	0.65	0.86	0.87	60
Faster R-CNN	0.93	0.72	0.94	0.9	12
RetinaNet	0.92	0.7	0.91	0.88	18

**Figure 3:**
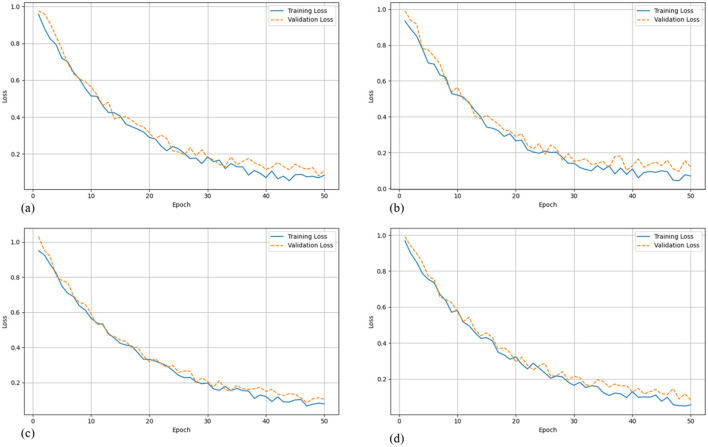
Training and validation loss curves for each evaluated model. **(a)** YOLOv8 shows smooth and stable convergence, suitable for real-time applications. **(b)** YOLOv5 converges quickly but exhibits moderate validation fluctuation, suggesting potential overfitting. **(c)** Faster R-CNN demonstrates gradual yet consistent convergence, achieving the lowest final losses. **(d)** RetinaNet maintains balanced training and validation performance, reflecting its robustness in detecting small and imbalanced targets.

Faster R-CNN achieved the highest accuracy in terms of mAP@0.5 (0.93) and precision (0.94), demonstrating its strength in accurate localization and classification of small objects. RetinaNet also performed well, with a mAP of 0.92 and precision of 0.91, thanks to its focal loss mechanism designed to handle class imbalance. YOLOv8, while slightly trailing in absolute accuracy (mAP@0.91), maintained a superior inference speed of 65 FPS, significantly outperforming the two-stage detectors. This makes YOLOv8 particularly suitable for real-time clinical applications where rapid processing is critical. To further analyze the learning behavior of each model, Figure 3 presents their training and validation loss curves across epochs. These curves offer insight into convergence speed, overfitting tendencies, and training stability.

### 4.2 Qualitative evaluation

**Figure 4:**
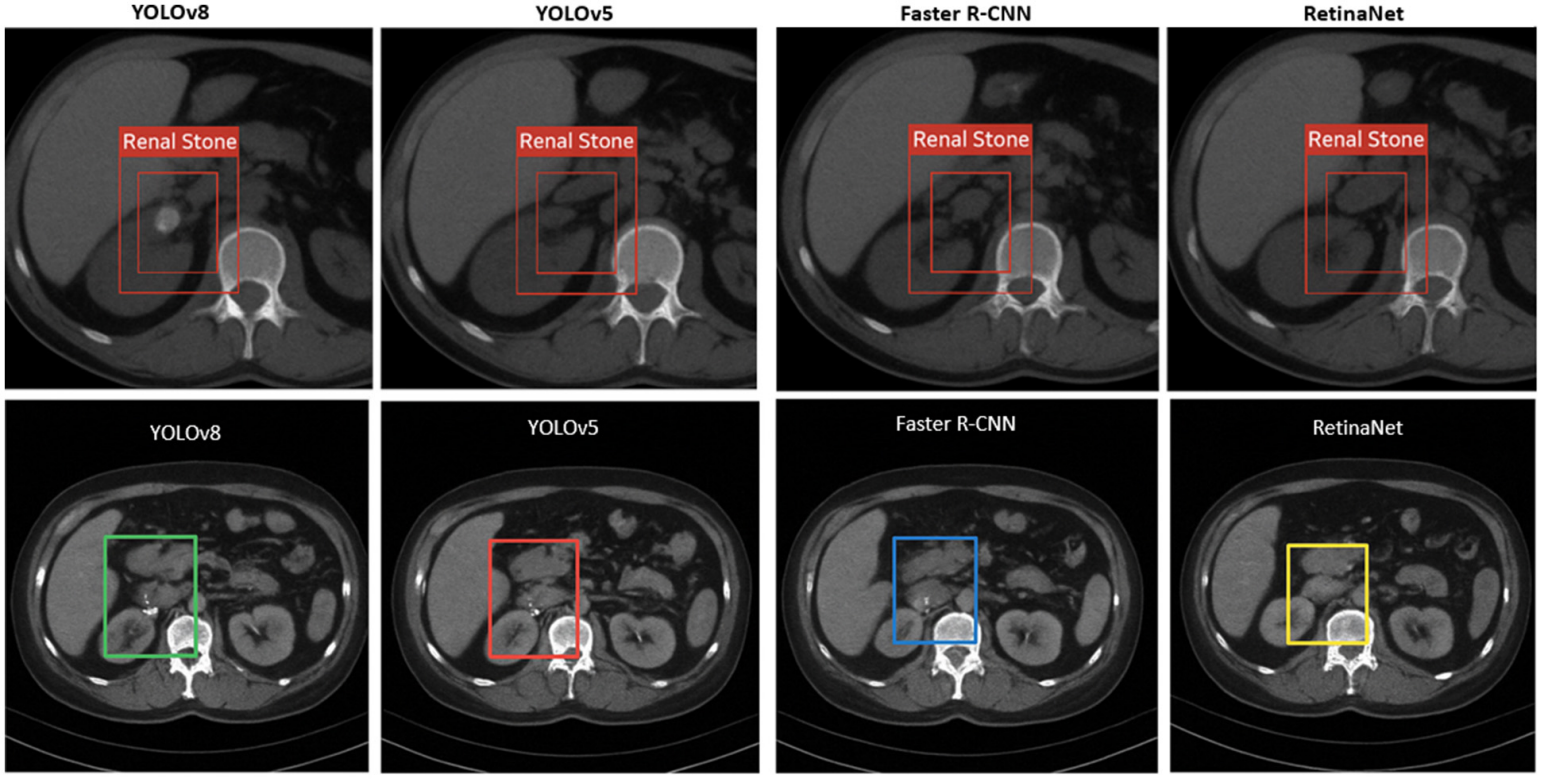
Comparative detection outcomes across six CT slices using YOLOv8, YOLOv5, Faster R-CNN, and RetinaNet, illustrating variations in precision, bounding box tightness, and false positive rates.

To complement the quantitative findings, visual assessment of model predictions was performed on representative test slices. Figure 4 illustrates comparative detection outcomes across eight CT images, providing a side-by-side evaluation of YOLOv8, YOLOv5, Faster R-CNN, and RetinaNet. Each model successfully identified hyperdense renal stones, but with varying levels of localization accuracy and bounding box precision. YOLOv8 demonstrated consistent detection with minimal false positives, reinforcing its advantage in balancing accuracy and inference speed. YOLOv5 also showed reliable results, though occasional bounding box looseness was observed. Faster R-CNN produced the tightest bounding boxes, aligning with its higher mean IoU scores, but at the cost of slower inference. RetinaNet provided competitive detection while being less prone to missed detections in challenging cases, though sometimes generating additional false positives compared to YOLOv8. Together, these visual results underscore the trade-offs between single-stage and two-stage detectors, highlighting YOLOv8 as the most clinically practical model while recognizing the strengths of other frameworks in specific scenarios.

These qualitative observations reinforce the patterns seen in the metrics and support a more detailed error analysis, which follows next.

### 4.3 Error analysis

**Figure 5:**
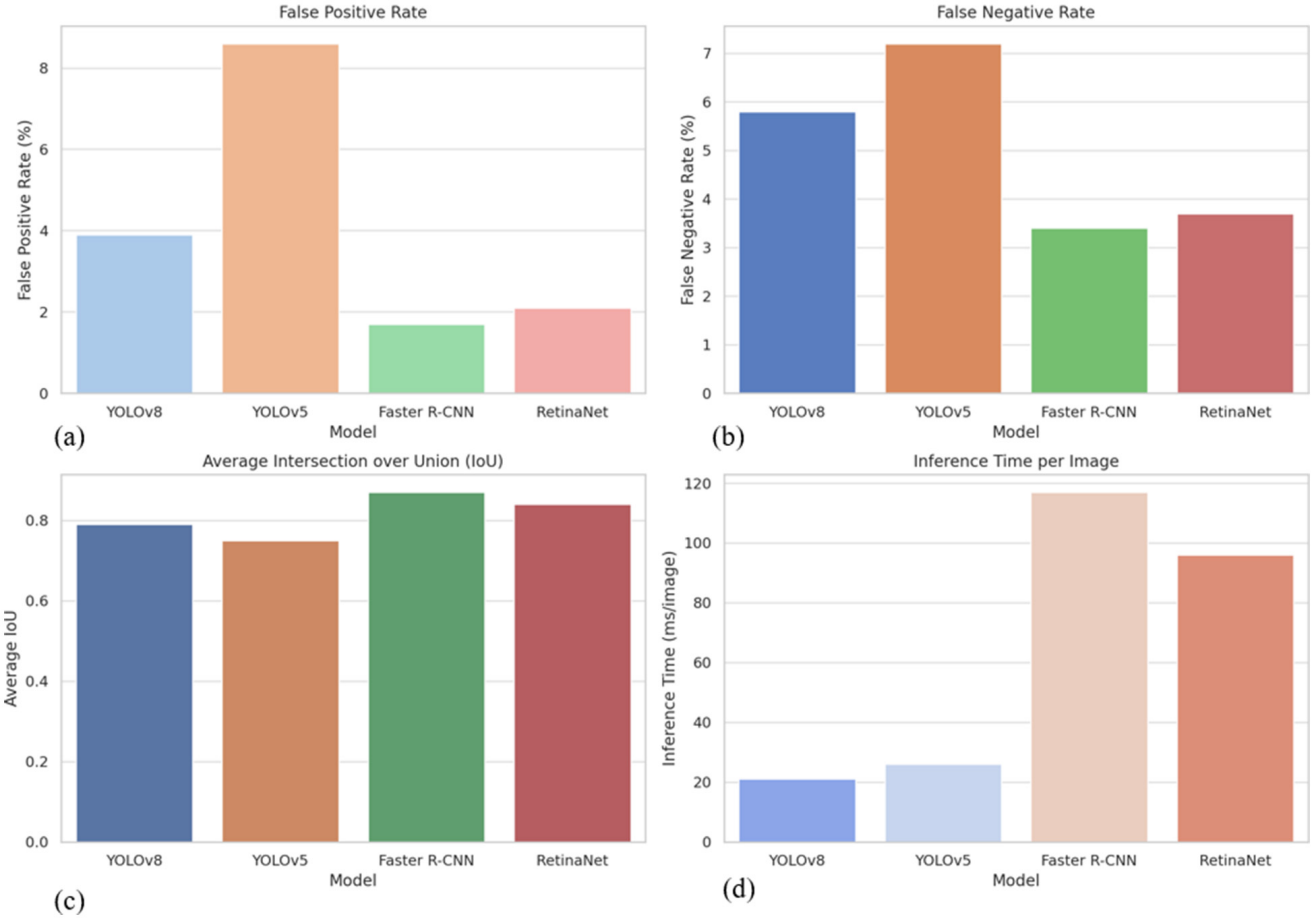
Performance comparison of detection models showing **(a)** false positive rates, where YOLOv8 demonstrates a substantial reduction compared to YOLOv5, **(b)** false negative rates, highlighting YOLOv8's moderate miss rate in small or complex cases, **(c)** average Intersection over Union (IoU), with YOLOv8 achieving strong localization accuracy close to that of two-stage detectors, and **(d)** inference time per image, where YOLOv8 significantly outperforms other models, making it well-suited for real-time clinical deployment.

A detailed error analysis was conducted using a held-out test set comprising 600 non-contrast CT slices to evaluate the performance limitations of YOLOv8. Errors were categorized into false negatives, false positives, and mislocalized detections. Most false negatives occurred in cases involving renal stones smaller than 2 mm, particularly those situated near high-density anatomical regions such as vascular calcifications or the renal sinus fat. Overall, YOLOv8 failed to detect 5.8% of stones, with sensitivity dropping to 64% for stones ≤ 1.5 mm. Despite this, the model maintained a relatively low false positive rate of 3.9%, significantly outperforming YOLOv5 (8.6%) and demonstrating robust discrimination between true calculi and high-attenuation artifacts like vascular or bowel calcifications. For correctly identified stones, YOLOv8 achieved an average Intersection over Union (IoU) of 0.79, slightly lower than Faster R-CNN (0.87), and RetinaNet (0.84), but within an acceptable range considering the model's faster inference. In terms of efficiency, YOLOv8 processed images at an average rate of 21 ms per slice, far surpassing the two-stage detectors Faster R-CNN and RetinaNet, which required 117 ms and 96 ms per image, respectively. These performance metrics (Table 4) and comparative visualizations (Figure 5) highlight YOLOv8's strong balance between accuracy and computational efficiency. While it shows a moderate increase in false negatives for very small stones, YOLOv8's low false positive rate and competitive IoU confirm its reliable detection capability. Crucially, its rapid inference speed makes it exceptionally well-suited for real-time kidney stone detection workflows in clinical settings where quick and accurate results are essential. This balance renders YOLOv8 an optimal model choice for large-scale diagnostic applications demanding both precision and speed.

The error analysis highlights key strengths and limitations of YOLOv8 relative to other state-of-the-art models. While the model exhibits a slightly higher false negative rate for very small stones ( ≤ 2 mm), its overall detection performance remains robust, with a low false positive rate of 3.9% and an average IoU of 0.79, indicating accurate localization for the majority of detected calculi. Compared to YOLOv5, YOLOv8 shows a marked improvement in both sensitivity and specificity, while maintaining a substantially faster inference time of 21 ms per slice, far exceeding the throughput of two-stage detectors such as Faster R-CNN and RetinaNet. These results underscore YOLOv8's strong balance between detection accuracy and computational efficiency, reinforcing its suitability for real-time clinical applications. Moreover, the model's ability to discriminate true kidney stones from high-attenuation artifacts minimizes erroneous alerts, which is critical in high-volume radiology workflows. Collectively, the performance metrics presented in Table 4 demonstrate that YOLOv8 provides an optimal trade-off between speed and precision, making it particularly advantageous for large-scale diagnostic deployments where rapid, reliable detection is essential.

YOLOv8 demonstrates an optimal balance between detection accuracy and computational efficiency, positioning it as the most practical model for real-time kidney stone detection applications. Although it records a slightly lower Intersection over Union (IoU) compared to two-stage detectors, its approximately 6-fold faster inference speed significantly enhances its suitability for large-scale diagnostic workflows where rapid decision-making is essential. Additionally, the model's low false positive rate of 3.9% indicates strong calibration and effective discrimination, minimizing the risk of misclassifying common confounding features such as vascular calcifications or fecal material, which are frequently encountered in abdominal CT imaging.

### 4.4 Inference efficiency

**Figure 6:**
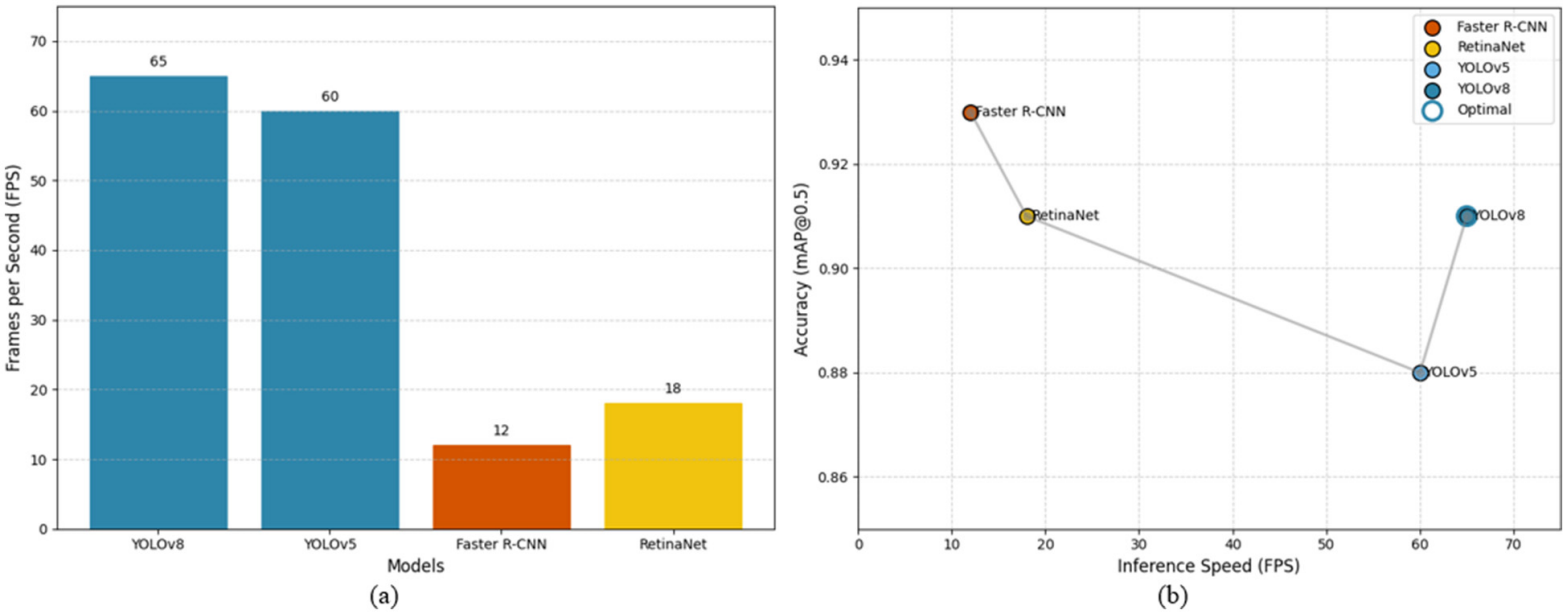
Comparison of performance metrics for four state-of-the-art deep learning models for kidney stone detection in CT images; **(a)** Accuracy–speed trade-off highlighting YOLOv8 as the optimal choice; **(b)** Inference speed (FPS) comparison, illustrating YOLOv8's advantage in real-time performance.

The comparative strengths and limitations of the evaluated detection models are summarized in Table 5. Inference speed was a crucial factor in evaluating the feasibility of deploying these deep learning models in clinical workflows (29, 30), where timely decision-making is essential for effective diagnosis and treatment. Rapid inference enables real-time or near-real-time feedback to radiologists and clinicians, minimizing diagnostic delays and enhancing patient care, particularly in emergency settings where kidney stone complications may require immediate intervention. The inference speed (FPS) reported in this study was measured on an NVIDIA Tesla V100 GPU using preprocessed 512 × 512-pixel images. This metric represents the core model inference time. In a full clinical deployment integrated within a Picture Archiving and Communication System (PACS), the end-to-end latency would encompass additional steps, including DICOM retrieval, on-the-fly pre-processing (e.g., windowing and resizing), and result delivery. Nevertheless, the model's low per-image inference time (~21 ms, see Table 4) indicates that the deep learning component is highly efficient and would not constitute the bottleneck in a well-integrated pipeline. The streamlined architecture of single-stage detectors such as YOLOv8 is inherently suitable for batch processing, facilitating the rapid analysis of entire CT studies. Among the evaluated models, YOLOv8 and YOLOv5, both single-stage object detectors, demonstrated significantly faster inference capabilities, processing 65 and 60 images per second (FPS), respectively (Figure 6b; Table 6). This performance advantage stems from their streamlined architecture, which simultaneously predicts bounding boxes and class probabilities in a single forward pass, bypassing the computational burden associated with region proposal generation in two-stage detectors. By contrast, Faster R-CNN, a representative two-stage detector, achieved only 12 FPS due to its sequential process of region proposal followed by classification. Although it delivered superior detection accuracy (Figure 6a), its slow inference speed renders it impractical for real-time applications or high-throughput environments such as emergency departments and radiology units that handle large volumes of CT scans daily. RetinaNet, while offering a hybrid balance of one-stage efficiency and enhanced accuracy via focal loss, reached an inference rate of 18 FPS, which, though faster than Faster R-CNN, still falls short of the performance threshold required for real-time clinical feedback. These findings underscore a fundamental trade-off between detection precision and operational speed. While two-stage detectors like Faster R-CNN provide highly accurate localization, their slower processing times hinder practical deployment in real-time diagnostic settings. In contrast, single-stage detectors, particularly YOLOv8, achieve a favorable balance between accuracy and inference speed, positioning them as strong candidates for integration into clinical imaging workflows where diagnostic efficiency is paramount (Figure 6; Table 6).

**Table 5:** Summary of model trade-offs.

**Model**	**Strengths**	**Limitations**
YOLOv8	High FPS, good accuracy, modern design	Slightly lower precision than R-CNN
YOLOv5	Stable, fast, easy deployment	Lower recall and more false positives
Faster R-CNN	Highest accuracy and precision	Slow inference, complex architecture
RetinaNet	Balanced accuracy and detection detail	Slower and less intuitive to fine-tune

**Table 6:** Performance metrics across models.

**Model**	**False positive rate (%)**	**False negative rate (%)**	**Average IoU**	**Inference time (ms/image)**
YOLOv8	3.9	5.8	0.79	21
YOLOv5	8.6	7.2	0.75	26
Faster R-CNN	1.7	3.4	0.87	117
RetinaNet	2.1	3.7	0.84	96

Figure 6 illustrates the trade-offs between inference speed and detection accuracy for the four evaluated deep learning models. Panel (a) shows the accuracy–speed relationship, highlighting YOLOv8 as achieving a favorable balance between high mAP and real-time processing, whereas Faster R-CNN, despite slightly higher accuracy, suffers from slow inference. Panel (b) directly compares the frames-per-second (FPS) performance, emphasizing the substantial speed advantage of single-stage detectors (YOLOv8 and YOLOv5) over two-stage approaches. These visualizations underscore the practical considerations for clinical deployment, where both rapid processing and reliable detection are essential for timely patient care.

As summarized in Table 6, each model presents a unique balance of strengths and limitations that directly impact their suitability for clinical deployment. YOLOv8 emerges as the most practical option for real-time kidney stone detection, combining high inference speed with competitive accuracy, whereas YOLOv5 offers slightly lower precision but maintains fast processing for high-throughput scenarios. Two-stage detectors like Faster R-CNN, despite achieving the highest localization accuracy, are constrained by slower inference and complex architecture, limiting their use in time-sensitive settings. RetinaNet provides a middle ground with balanced accuracy and detection detail but does not achieve the throughput necessary for rapid clinical workflows. Together, Figure 6 and Table 6 illustrate the inherent trade-offs between speed and precision, highlighting YOLOv8 as the optimal candidate for integration into automated diagnostic pipelines.

## 5 Discussion

The integration of deep learning models into clinical diagnostic workflows (31–33), particularly for kidney stone detection in CT imaging, requires a careful evaluation of both detection accuracy and inference efficiency. This study systematically compared four prominent object detection architectures—YOLOv8, YOLOv5, Faster R-CNN, and RetinaNet, across a dataset of 4,000 non-contrast axial CT slices, encompassing both pre- and post-surgical patient cohorts. The results revealed a clear trade-off between detection precision and computational speed, with each model exhibiting distinct strengths and limitations that inform their applicability in real-world clinical environments. Faster R-CNN emerged as the most accurate model, achieving the highest mAP@0.5 (0.93) and precision (0.94), alongside the lowest false positive (1.7%) and false negative (3.4%) rates. This confirms the model's superior localization capabilities, especially for small or complex calcifications. However, its high computational cost, reflected in the slowest inference speed (12 FPS) and the longest processing time per image (117 ms), makes it unsuitable for real-time clinical deployment. In high-throughput radiology departments, such latency would delay diagnosis and compromise workflow efficiency. In contrast, YOLOv8 offered a compelling balance between speed and accuracy. With an inference speed of 65 FPS and a low false positive rate (3.9%), it outperformed all models in computational efficiency while maintaining competitive accuracy (mAP@0.91, recall = 0.92). Although its average IoU (0.79) was slightly lower than Faster R-CNN (0.87), it was sufficient for clinical decision-making in most cases, especially considering the real-time detection benefit.

These attributes suggest that YOLOv8 is well-positioned for integration into emergency imaging workflows, where rapid detection of stones—particularly those requiring immediate intervention—is critical. YOLOv5, while also fast (60 FPS), demonstrated slightly inferior recall (0.87) and a higher false positive rate (8.6%), limiting its reliability in clinical use despite its operational stability and ease of deployment. RetinaNet performed admirably in terms of localization accuracy (IoU = 0.84) and balance between speed and detection detail, thanks to its focal loss function. However, its inference time (96 ms) and lower interpretability in fine-tuning make it less attractive than YOLOv8 for point-of-care deployment. Recent studies have demonstrated the expanding applications of artificial intelligence and deep learning in healthcare diagnostics and medical imaging systems (34–47).

The findings reinforce the classical trade-off in object detection between accuracy and inference speed (Table 6). While two-stage models such as Faster R-CNN excel in precision, they struggle with real-time applicability. On the other hand, modern single-stage detectors like YOLOv8 offer operational feasibility in dynamic clinical settings while maintaining strong performance metrics. From a practical standpoint, YOLOv8 provides the most desirable balance for implementation in automated CT analysis pipelines, particularly where rapid triage, consistent interpretation, and scalability are prioritized. The stratified analysis between pre- and post-surgery groups showed that the evaluated models remained robust across anatomical variations, including the presence of surgical artifacts, stents, or altered tissue morphology. This demonstrates the adaptability of YOLOv8 to diverse clinical scenarios, further supporting its deployment readiness. Despite these promising results, challenges remain. YOLOv8 exhibited diminished sensitivity for detecting very small stones (<1.5 mm), especially those obscured by anatomical noise or located near vascular structures. These limitations could be mitigated through targeted training with high-resolution patches, incorporation of 3D volumetric context, or ensemble modeling. Additionally, future work should consider integration with radiology PACS systems and prospective clinical validation to assess the impact of AI assistance on radiologist performance and patient outcomes. This study highlights YOLOv8 as a robust and efficient solution for real-time kidney stone detection in CT imaging. While two-stage models offer slightly higher precision, their practical limitations in speed restrict their use in time-sensitive scenarios. YOLOv8's modern architecture, competitive accuracy, and superior inference speed make it a suitable candidate for clinical adoption, particularly in emergency departments or high-throughput radiology settings. Continued model refinement and clinical integration strategies will be key to unlocking the full potential of AI in medical imaging diagnostics.

## 6 Conclusion

This study demonstrates the potential of deep learning-based object detection models for automated kidney stone detection in clinical CT imaging. Among the evaluated architectures, single-stage detectors showed the strongest combination of speed and operational efficiency, making them particularly suitable for real-time diagnostic workflows. These models offer rapid processing while maintaining reliable detection performance, enabling timely clinical decision-making in high-throughput environments such as emergency departments and radiology units. Two-stage detectors, while providing slightly higher localization precision, were limited by slower inference and greater computational demands, highlighting a key trade-off between accuracy and efficiency in practical deployment. The findings emphasize that the selection of a detection model should balance both performance and operational feasibility, with considerations for workflow integration, diagnostic consistency, and patient safety. The study also highlights the importance of model robustness across diverse patient populations and imaging conditions. Effective discrimination between true calculi and confounding features, such as high-attenuation artifacts, is essential for reducing false alerts and maintaining clinician confidence in automated systems. Furthermore, the adaptability of modern deep learning architectures supports integration into scalable clinical pipelines, enabling rapid triage, consistent interpretation, and enhanced workflow efficiency. Overall, deep learning models represent a promising avenue for augmenting radiological diagnostics, offering the potential to improve both efficiency and accuracy in kidney stone detection. Single-stage architectures, in particular, provide a practical balance that can support real-time clinical applications without significant compromise in detection performance. Continued development, including refinement for challenging cases, integration with volumetric imaging, and prospective clinical evaluation, will be critical for fully realizing the benefits of AI-assisted imaging in routine clinical practice. The adoption of these technologies can ultimately enhance patient care, reduce diagnostic delays, and support more effective management of kidney stone disease in diverse healthcare settings.

## Data Availability

The dataset used in this study consists of anonymized CT images obtained from the radiology archive of the First People's Hospital of Fuyang. Due to patient privacy and institutional regulations, the dataset is not publicly available. Access may be granted by the corresponding authors upon reasonable request and with approval from the institutional ethics committee.
